# The Potential Role of Herpes Simplex Virus Type 1 and Neuroinflammation in the Pathogenesis of Alzheimer's Disease

**DOI:** 10.3389/fneur.2021.658695

**Published:** 2021-04-06

**Authors:** Kathlyn Laval, Lynn W. Enquist

**Affiliations:** Department of Molecular Biology, Princeton University, Princeton, NJ, United States

**Keywords:** neuroinflammation, Alzheimer's disease, pathogenesis, peripheral nervous system, herpesvirus

## Abstract

Alzheimer's disease (AD) is a neurodegenerative disease affecting ~50 million people worldwide. To date, there is no cure and current therapies have not been effective in delaying disease progression. Therefore, there is an urgent need for better understanding of the pathogenesis of AD and to rethink possible therapies. Herpes simplex virus type 1 (HSV1) has recently received growing attention for its potential role in sporadic AD. The virus is a ubiquitous human pathogen that infects mucosal epithelia and invades the peripheral nervous system (PNS) of its host to establish a reactivable, latent infection. Upon reactivation, HSV1 spreads back to the epithelium and initiates a new infection, causing epithelial lesions. Occasionally, the virus spreads from the PNS to the brain after reactivation. In this review, we discuss current work on the pathogenesis of AD and summarize research results that support a potential role for HSV1 in the infectious hypothesis of AD. We also highlight recent findings on the neuroinflammatory response, which has been proposed to be the main driving force of AD, starting early in the course of the disease. Relevant rodent models to study neuroinflammation in AD and novel therapeutic approaches are also discussed. Throughout this review, we focus on several aspects of HSV1 pathogenesis, including its primary role as an invader of the PNS, that should be considered in the etiology of AD. We also point out some of the contradictory data and remaining knowledge gaps that require further research to finally fully understand the cause of AD in humans.

## Introduction

Alzheimer's disease (AD) is one of the most common neurodegenerative diseases of humans with 50 million people affected worldwide (1). The disease was first described by a German psychiatrist and neuropathologist, Alois Alzheimer, in 1907. He reported a peculiar severe disease process of the cerebral cortex in a 50-year-old woman, named Auguste D, admitted to a psychiatric hospital for paranoia, progressive sleep and memory disturbance, confusion, and aggression (2). Clinically, AD begins with mild symptoms of memory loss and slowly leads to global cognitive impairment. The cognitive deficits are frequently accompanied by severe neurological and psychiatric symptoms in late-stage disease (3, 4). The most relevant AD risk factor is age, with the prevalence of AD rising exponentially after 65 years of age, with one in ten individuals affected (5). Early-onset AD (EOAD) are usually diagnosed before age 65 while late-onset AD (LOAD) develops after age 65. Approximately 95% of all AD cases are LOAD while a few percentages are attributed to EOAD. The LOAD is mainly sporadic with a complex multifactorial etiology (e.g., age, environmental and genetic factors, pathogens). In contrast, the EOAD is almost entirely genetic disease with 92–100% heritability (6–10). AD represents a growing public health burden as populations live longer. Indeed, epidemiological studies suggest that the overall prevalence of AD is expected to double within the next 20 years (11). On average, a person can live 4–8 years after diagnosis and in rare cases, up to 20 years. However, patients require total care in the later stages of the disease. Therefore, AD has also a significant financial impact on global healthcare systems. AD care costs have been predicted to be around $2 trillion in 2030 with ~75 million people affected worldwide if effective interventions are not found (12). So far, there is no cure and current therapies have not been effective in delaying progression. Donepezil, a cholinesterase inhibitor and FDA-approved drug, is currently the most common treatment for mild to severe forms of AD. This drug enhances acetylcholine availability and increases neuronal transmission in the brain by inhibiting its breakdown in the synapse. This treatment shows modest benefit in cognitive function and daily activities of some AD patients compared to placebo treatment (13). However, Donepezil presents severe adverse side-effects and patients who discontinue this drug experience accelerated cognitive decline (14). Consequently, there is an urgent need to gain a better understanding of the pathophysiology of AD in order to develop effective therapies.

## The Pathogenesis of AD

The two neuropathological hallmarks of AD are the extracellular deposits of amyloid-β (Aβ) peptide and the intracellular accumulation of hyperphosphorylated tau protein in the brain. These pathologic features are commonly accompanied by the loss of neurons and synapses and reactive gliosis. Aβ peptide is produced by the sequential, proteolytic processing of the amyloid precursor protein (APP). APP is produced primarily by neurons and is transported anterograde in axons to synapse terminals (15). While its function remains largely unclear, APP has been suggested to play a role in synapse formation, synaptic transmission and neurogenesis (16, 17). APP has demonstrated neuroprotective properties following traumatic brain injury (18). However, its processing and cleavage represent the foundation of the “amyloid cascade hypothesis” in which, the accumulation of neurotoxic Aβ by-products in neurons causes AD (19, 20). APP processing can occur through 2 distinct pathways (21). In the non-amyloidogenic pathway, APP cleavage is mediated by the α-secretase and γ-secretase and results in the production of 3 fragments: a secreted C-terminal fragment (sAPP-α), p3, and the APP intracellular domain. In the amyloidogenic pathway, APP is cleaved by the β-secretase, which generates a large soluble extracellular secreted domain (sAPP-β). The remaining APP bound fragment is cleaved multiple times by the γ-secretase to produce neurotoxic Aβ peptides, variable in length, including Aβ-40 and Aβ-42 fragments. Genetic mutations in APP and the γ-secretase complex [presenilin-1 (PSEN1) and PSEN2] have been reported to induce aberrant APP processing and overproduction of Aβ in the brain and cause EOAD (9, 22, 23). Several lines of evidence suggest that the impaired clearance of Aβ rather than its overproduction leads to the accumulation of Aβ and amyloid deposition in the brain (24). The clearance of Aβ in the brain can be accomplished by several mechanisms, including non-enzymatic and enzymatic pathways. The non-enzymatic pathway consists of: (1) the interstitial fluid drainage of Aβ; (2) the uptake of Aβ by microglial phagocytosis and transport into the blood circulation; (3) the autophagy in microglia and further degradation of Aβ fibrils *via* the lysosomal system; (4) transcytosis of Aβ across the blood-brain barrier via the low-density lipoprotein receptor family (25–29). The enzymatic pathway comprises the activity of Aβ-degrading enzymes such as neprilysin, insulin-degrading enzyme, matrix metalloproteinase-9, and glutamate carboxypeptidase II (30). These mechanisms are not covered in this review as several excellent reviews have addressed these topics in more detail (31–33).

The aggregation of Aβ proteins into plaques enhances the formation of neuro-fibrillary tangles (NFT), which consist of the hyperphosphorylation, misfolding, and aggregation of tau protein, a microtubule associated protein normally located in axons. There are multiple ways by which NFT may damage neurons and glial cells in the brain (34). For instance, by acting as physical barriers in the cytoplasm, compromising normal cellular functions, and homeostasis through inhibition of proteasomal activity (35). NFT may also cause neuronal toxicity by reducing normal tau functions such as tau-mediated regulation of microtubule dynamics (36). Ultimately, NFT are able to self-propagate, spread to synapses to promote synaptic and cognitive dysfunction in AD. While the toxic gain of function and the loss of normal tau functions are thought to play a role in inducing neuronal death and synaptic loss, their underlying mechanisms are still largely unclear.

Aberrant APP processing and overproduction of Aβ can promote EOAD, but it is not clear whether these events can also cause LOAD. Most of the previous AD research has supported the amyloid cascade hypothesis and focused on identifying the mechanisms underlying Aβ plaque deposition in LOAD. However, this hypothesis has been the source of increased controversy over the years (37, 38). For instance, the presence of Aβ deposition has been detected in brains of elderly normal individuals, suggesting that Aβ plaques might be non-toxic and related to aging (39, 40). In fact, treatments aimed at targeting and reducing Aβ protein levels have largely failed in clinical trials (41, 42). Several alternative approaches for understanding AD pathogenesis are now being considered. For example, if the accumulation of Aβ in the brain occurs 10–20 years before the onset of cognitive impairment, early interventions could be more successful than those given to people with late-stage disease (43). An alternative view is the tau hypothesis, in which tau abnormalities are as important if not more than Aβ for disease progression (44). Because the role of Aβ accumulation and aggregation as a primary cause of LOAD remains debatable, there is an urgent need to investigate the pathogenesis of AD from a new angle and to rethink possible therapeutic strategies.

## Herpes Simplex Virus Type 1 (HSV1): A Potential Player in the Infectious Hypothesis of AD

In the last years, the inflammatory-infectious hypothesis of AD has gained support as an alternative to the amyloid hypothesis that has dominated the AD field for decades. Several infectious agents, including bacteria and viruses have been implicated in the pathogenesis of AD. Human herpes simplex virus type 1 (HSV1) stands out as a possible significant player (45–50). Numerous publications have linked evidence for HSV1 infection with the development of sporadic AD (see section The Involvement of HSV1 in AD). It was proposed that reactivation of latent HSV1 infections may cause local neuronal damage and inflammation, which over time may lead to the deposition of Aβ and abnormal phosphorylation of tau in the brain. However, these data are correlative and do not demonstrate a direct causal relationship between HSV1 and AD. In this section, we aim to provide an understanding of the pathogenesis of HSV1 with a focus on HSV1 reactivation and latency in humans and *in vivo* models. This knowledge is essential as we discuss the most relevant studies looking into the role of HSV1 in the pathogenesis of AD.

### The Pathogenesis of HSV1

HSV1 is a neuroinvasive pathogen, which commonly causes mucosal lesions, infectious blindness (herpes keratitis), as well as rare cases of encephalitis in humans. HSV1 infection is widespread around the world, with seropositivity among older adults (>65 years old) estimated to be 60–90% (51, 52). After primary replication in the mucosal epithelia, HSV1 reaches neurons of peripheral nervous system (PNS) that innervate the infected tissue. In the PNS, active replication does not occur and a lifelong latent infection is established in peripheral sensory and autonomic neurons [e.g., trigeminal ganglia (TG) and ciliary ganglia]. These quiescent infections may reactivate periodically (53, 54). Upon reactivation, newly replicated virus particles travel back to the mucosal epithelium. Secondary replication in the mucosal epithelium can cause local lesions in a subset of individuals while asymptomatic shedding can occur in others (55, 56). HSV1 can also infect the central nervous system (CNS) from peripheral infections and cause HSV encephalitis (HSE). While HSE is the most common fatal sporadic encephalitis in humans, its incidence is relatively rare with ~2–4 cases/1,000,000 people worldwide (57, 58).

Several studies demonstrated the presence of HSV1 DNA in postmortem brain tissues from asymptomatic patients by *in situ* hybridization and polymerase chain reaction (PCR) (59–63). The exact mechanism by which HSV1 gains access to the CNS in humans is unclear. The most likely route includes HSV1 retrograde transport to the brainstem via the trigeminal nerve that innervates the orofacial or corneal epithelium to the brainstem (64). An alternative route may involve viral spread via the olfactory tract (65). Direct CNS infection from the periphery as well as the possibility of reactivation of latent HSV1 within the brain are also considered but these possibilities are still an area of research and debate (66). Some studies with mice are informative. For instance, in the *in vivo* study of Kastrukoff et al., 10 mice were inoculated via the oral mucosa using a scarification method. The presence of HSV1 DNA and antigen positive cells in the brain were determined by *in situ* hybridization and immunohistochemistry (IHC) techniques, respectively (67). The authors demonstrated that HSV1 DNA and antigen positive cells were detected sequentially in the TG and throughout the brain as the infection progressed (first, the brainstem followed by the cerebellum and then the cerebral hemispheres). These areas are widely separated from each other and imply spread via neuronal circuitry. These findings are in accordance with previous reports on the spread and localization of HSV1 from the PNS to the CNS (68, 69). Interestingly, the authors also found “focal” areas of HSV1 antigen positive neuronal and glial cells. The authors showed that despite the clearance of viral antigen positive cells over time, HSV1 DNA was detected in the mouse brain 25 days after inoculation. This finding implies that a latent HSV1 infection was established in the brain. Indeed, the subsequent reactivation of HSV1 from these localized sites of CNS latent infection may cause sub-clinical or mild encephalitis in mice. Such processes, which may occur sporadically, would be difficult or impossible to detect or diagnose in asymptomatic patients.

Two *ex vivo* studies demonstrated that infectious HSV1 could be recovered from dissociated mouse TG as well as brain explants (70, 71). The authors found that following eye inoculation with HSV1 KOS and the more virulent McKrae strain, 71% and 80% of brainstem samples reactivated latent HSV1 at 30 days post-infection, respectively. A higher percentage of TG samples (100 and 90%) reactivated latent HSV1. Using the hyperthermia reactivation model, Yao et al. detected higher levels of HSV1 genomes in the brainstem of mice latently infected with HSV1 294.1 and McKrae strains than those in the TG (72). Moreover, HSV1 294.1 and McKrae reactivations occurred earlier in the mouse brainstem and TG (13 and 16 h after hypothermic stimulation). The authors suggested that the poor viability of brain explant cultures might explain the discrepancy in reactivation sites observed between *ex vivo* and *in vivo* results. A recent *in vivo* study by Doll et al. reported contradictory results. These authors found more infectious HSV1 particles and a higher frequency of viral reactivation in the TG compared to the brainstem of latently infected mice (73). In addition, HSV1 antigen was detected in the TG, but not the brainstem of these animals, 24 h after post-reactivation. Overall, these studies confirm that HSV1 can establish latency in the PNS and CNS after peripheral infection. They also suggest that HSV1 spreads to the CNS after reactivation from latency in the PNS. These studies point out the importance of initial infection of the PNS and possible effects in the brain that might have implications for AD research.

### The Involvement of HSV1 in AD

In the early 1990's, two studies from Jamieson et al. first demonstrated that HSV1 DNA is present in a higher proportion of brains of elderly people with or without dementia compared to young people (60, 61). Additional studies showed that HSV1 DNA is detected in more AD brain samples than non-AD ones (74, 75). PCR results specifically correlated HSV1 DNA with β-amyloid deposits in the cerebral cortex. HSV1 antigens were also detected in the cytoplasm of cortical neurons of AD brain samples by IHC. However, these and other studies do not prove that HSV1 DNA is directly associated with Aβ plaques in the brain. On the one hand, 72% of HSV1 DNA was associated with β-amyloid plaques in AD brains compared to 24% in aged normal brains (76, 77). On the other hand, another study showed that only 55% of HSV1 DNA is plaque-associated in AD brains (63). Differences in the methodology used between the two studies (*in situ* PCR and immunohistochemistry vs. TaqMan PCR and serology) and sample sizes might explain the difference between results.

In the late 2000's, a population-based cohort study showed that the risk of AD increased in elderly subjects correlated with positive titers of anti-HSV1 IgM antibodies. These antibodies are indicators of primary infection or recent reactivation and replication of the virus (78). The authors suggested that the presence of anti-HSV1 IgM antibodies is highly correlated with incidence of AD. These findings were consistent with those of Féart et al., who correlated anti-HSV1 antibodies and plasma levels of Aβ_1−40_ and Aβ_1−42_ (two biomarkers of the disease) in a large study sample (79). This work demonstrated that high IgM levels, but not IgG levels, are significantly associated with low plasma levels of Aβ_1−40_ and Aβ_1−42_. The authors proposed that HSV1 reactivation leads to the accumulation of Aβ-deposits in the brain and subsequent decrease of Aβ plasma levels. Another HSV1 antibody study, by Lovheim et al. involved a larger number of participants (*N* = 3,432) with a longer follow-up (11 years). This study confirmed the presence of anti-HSV1 IgM antibodies at baseline in serum samples and almost doubled the risk for AD (80). A recent systematic review and meta-analysis further compiles up-to-date evidence that HSV1 is a major risk factor for AD (81).

In the last decade, several studies and animal models have been developed to determine the specific role of HSV1 reactivation in the progression of AD (82). Using a mouse model of encephalitis, Martin et al. showed that HSV1 immediate early protein ICP4 is expressed in TG by 7 days after intranasal infection, and also in the cerebral cortex by 60-days post-infection (dpi) (83). The expression of the HSV1 ICP4 protein was accompanied by the increased expression in the brain of neuroinflammatory (toll-like receptor 4; TLR-4) and early neurodegenerative markers (phospho-tau and TauC3) in the brain. The authors suggested that the presence of this HSV1 protein in the brain at 60 dpi correlated with HSV1 reactivation from CNS latency sites and the induction of neuroinflammation and neurodegeneration. These data must be interpreted with caution because the correlation of HSV1 ICP4 expression in both TG and cerebral cortex at 60 dpi does not prove that HSV1 reactivated from CNS latency sites only. Perhaps, HSV1 reactivated from PNS or both PNS and CNS latency sites. Recently, De Chiara et al. developed a model of recurrent HSV1 in mice experiencing repeated cycles of viral reactivation (84). This study was the first to report a connection between HSV1 and AD in a long-term *in vivo* study. After labial HSV1 inoculation, infectious virus particles were detected in the TG and several brain regions of infected mice after 4 dpi. They showed that HSV1 replicated in the brain of infected mice in response to a reactivation stimulus. Moreover, the repeated HSV1 reactivations induced the accumulation of Aβ and deposition in amyloid plaques as well as increased levels of phospho-tau in the brain. In addition, repeated reactivations resulted in impairment of cognitive functions in infected mice. Note that Aβ and phosphor-tau levels were not evaluated in the TG following viral reactivation. It also was unclear if all of the infected, unstressed mice actually had HSV1 in the CNS, as approximately of 21% of mice were negative by PCR for the virus in the brain. These results should be validated in future studies. Overall, the consensus hypothesis to date is that repeated asymptomatic HSV1 reactivations can occur in the brain during episodes of stress or immunosuppression, which induces cumulative neuronal damage and AD pathology. It was also suggested that periodic HSV1 reactivations occur more frequently in elderly brains as the immune system declines with age (45). However, these studies focused primarily on HSV1 CNS reactivation and latency in the pathogenesis of AD. A simple question is how does HSV1 get to the CNS? It is well-known that HSV1 invades the PNS where it establishes latency and that the PNS is connected to the CNS. Perhaps the virus enters the CNS from the periphery by axonal transport. Future studies should clarify the role of reactivation events in the PNS and the subsequent contributions of these peripheral processes to the initiation and development of AD in the brain.

Recently, three major studies demonstrated a direct role of HSV1 in the pathogenesis of AD. Tzeng et al. reported that HSV1 infection in humans led to a significant risk of later development of AD and that treatment with specific herpes antiviral drugs at the time of infection markedly reduced that risk by a factor of 10 (85, 86). This study was performed over a 10-year period with starting participants older than 50 years who had a newly diagnosed HSV1 infection. These findings are in accordance with a previous *in vitro* study showing that anti-HSV1 agents, such as acyclovir, penciclovir and foscarnet reduced Aβ and tau accumulation in HSV1-infected Vero cells (87). Another study by Eimer et al. demonstrated that in 3D human neural immortalized cells, Aβ oligomers inhibited HSV1 infection and significantly protected transgenic mice from acute HSV1 encephalitis following intracranial inoculations (88). The authors proposed that Aβ is an intrinsic antiviral response of neurons. Recently, Cairns et al. developed a 3D-brain tissue model using human-induced neural stem cells (hiNSCs). This study was the first to demonstrate that HSV1 infection directly causes a remarkable AD-like phenotype within 1 week after infection (89). This 3D brain tissue model demonstrated multiple aspects of AD physiology, including Aβ fibrillar plaque-like formations upon HSV1 infection, neuronal loss, neuroinflammation, and reactive gliosis. These findings corroborate previous *in vitro* studies showing that HSV1 infection induces APP and the accumulation of Aβ and other neurotoxic APP fragments in human and rat CNS neuronal cells (90, 91). Most importantly, the study from Cairns et al. showed that treatment with valacyclovir, a common herpesvirus antiviral drug, significantly reduced the AD-like phenotype in infected hiNSCs.

Alpha herpesviruses like HSV1 have evolved a well-defined relationship with neurons. They have evolved to survive in their host with limited damage to neurons and to disseminate to other hosts by establishing a latent, reactivable infection in neurons. It has been proposed that neurons produce certain synaptic proteins (e.g., Aβ) as an ancient protective mechanism against viral infections. This phenomenon could justify the use of HSV1 antiviral treatments and HSV1 vaccines as clinical treatments of AD. Antiviral drug treatments have already shown promising results to reduce AD pathology *in vivo* and *in vitro*. The development of a HSV1 vaccine may represent another effective treatment. Other more general interventions to block or reduce viral infections may be useful. For example, a pilot study demonstrated that treatment of 42 AD patients with IFN-β slightly, but not significantly, reduced disease progression compared to control group (92). IFN-β was well-tolerated with limited adverse effects, which may warrant further investigation in larger studies.

## Neuroinflammation: At the Center of the Infectious Hypothesis of AD

Two critical elements of the infectious hypothesis are that the infectious agent must persist for years or periodically infect the host and that the immune response to this agent involves the nervous system. HSV1 infections meet these two requirements. It is a ubiquitous human infection that infects the nervous system and established a reactivable latent infection. Despite a strong immune response, the infection is not cleared and lasts for the life of the host.

Neuroinflammation is an important component of the infectious hypothesis of AD. It is defined as an inflammatory response in the nervous system to infection or injury. Neuroinflammation is considered a major driving factor in neurodegeneration and AD pathology, which starts early in the course of the disease, prior to the formation of Aβ plaques in the brain (93, 94). It may be triggered by infectious agents including viruses (95). Neuroinflammation may represent a promising drug target to prevent neurodegeneration and AD. In this section, we summarize key findings of the role of the neuroinflammation in the viral etiology of AD.

### The Neuroinflammation in the Pathogenesis of AD

Microglia, astrocytes and oligodendrocyte are the main glia cells in the brain (96). These cells control the microenvironment of CNS neurons and are crucial in synaptic remodeling, tissue repair, and neuronal survival following CNS injury (97–100). Neuroinflammation stimulates the local activation of glia cells surrounding damaged or infected neurons. Upon activation, microglia and astrocytes cells undergo a series of morphological and functional changes and acquire a “reactive” phenotype (101). For instance, activated microglia migrate to the site of infection/lesion and phagocytose cellular debris. In addition, activated glia cells release a wide range of proinflammatory mediators, including IL-1β, IL-6, TNF-α, aimed at preventing further damage to the CNS (102). Astrocytes can also be activated by the release of inflammatory mediators from microglia (103). The interaction between activated microglia and astrocytes plays a key role in the neuroinflammatory response (104, 105). While the activation of glia cells is part of a protective immune response to tissue injury or infections, its long-lasting and uncontrolled activation can be deleterious, causing chronic inflammation, and neurodegeneration.

In the early pathogenesis of AD, it has been suggested that microglia activation reduces the accumulation of Aβ in the brain by increasing its phagocytosis, clearance, and degradation (106, 107). Microglia bind soluble Aβ oligomers and fibrils via receptors, including class A scavenger receptor A1, toll-like receptors (e.g., TLR2, TLR4) and coreceptor CD14, and subsequently phagocytose and clear Aβ. However, the persistent microglia activation stimulated by the binding of microglia to Aβ can increase the production of inflammatory mediators and reactive oxygen species (ROS), which amplifies the neuroinflammatory response in the brain (108). Using transgenic PS1-APP mice, an established model of AD, Hickman et al. demonstrated that the continuous production of proinflammatory cytokines reduces the expression of the phagocytosis receptors expressed in microglia and alters their functions (109). The sustained activation of microglia cells has been shown to exacerbate both amyloid and tau pathology and may serve as a link in the pathogenesis of AD (110, 111). Indeed, the prolonged activation results in an impaired clearance of Aβ and increased accumulation in the CNS (112). Moreover, the increase of inflammatory cytokines expression contributes to tau hyperphosphorylation and neuronal loss. In particular, IL-6 has been shown to induce tau hyperphosphorylation in rat hippocampal neurons (113). Ultimately, the prolonged priming of microglia to an inflammatory state causes neuronal damage and neurodegeneration and contributes to the progression of AD.

Moreover, the activation of pattern recognition receptors (PRRs) expressed by microglia can influence the neuroinflammation and AD pathology. PRRs are either membrane-bound [e.g., Toll-like receptors (TLRs)] or intracellular, such as the nucleotide-binding domain and leucine-rich repeat-containing receptors (NLRs), AIM2-like receptors (ALRs), and the tripartite motif-containing (TRIM) family member pyrin (114). The sensing of pathogen-associated molecular patterns (PAMPs) and danger-associated molecular patterns (DAMPs) by cytosolic PRRs results in the assembly of the inflammasome (115). This multiprotein complex is involved in the initiation of inflammatory responses (e.g., production of interleukins IL-1β and IL-18) and in the induction of pyroptosis, a highly-pyrogenic inflammatory form of cell death (116, 117). Several inflammasomes, including the pyrin domain-containing 3 (NLRP3) inflammasome, have been shown to play a crucial role in the development and progression of Aβ plaque formation and tau-induced pathology (118–120). Interestingly, Ising et al. demonstrated that the accumulation and deposition of Aβ and NFT formation are detected by cytosolic PRRs, triggering NLRP3 inflammasome activation in microglia (118). This mechanism ultimately exacerbates the neuroinflammation and AD pathology.

A study from Wyss-Coray et al. demonstrated that reactive mouse astrocytes internalize and degrade Aβ, suggesting a neuroprotective role of these cells in AD (121). However, other studies showed that reactive astrocytes have neurotoxic properties and their persistent inflammatory phenotype contribute to the loss of functions (122, 123). Astrocyte dysfunction has been shown to result in an increase of pro-inflammatory mediators release, decrease of glutamate uptake, loss of synapses, and ultimately cognitive deficits in AD (124). A number of recent reviews further examine particular aspects of the role of microglia and astrocytes in the neuroinflammatory response in AD, including phenotypical and functional changes, genetic variants of the innate microglial immune receptor TREM2, which has been associated with an increased risk of LOAD (105, 125–129).

Several rodent models of neuroinflammation have been developed to study the inflammatory hypothesis of AD and to test potential new therapeutics. Ideally, these models should demonstrate early chronic neuroinflammation prior to the formation of Aβ plaques and NFT. An interesting model is polyI:C-induced neuroinflammation, which consists of the systemic injection of polyI:C, a synthetic double-stranded RNA that induces an innate immune analogous to an acute viral infection. Systemic injection of polyI:C on gestational day 17 mice increased IL-1 and IL-6 levels in the plasma and brains of treated animals compared to controls, starting from 3 weeks post-injection and was sustained throughout aging (130). This prenatal immune challenge also demonstrated a significant, age-dependent increase of tau hyperphosphorylation starting from 3 months of age and of APP, starting as late as 12 months of age. Cognitive impairment was detected in mice 20 months post-injection. Importantly, a second systemic immune challenge performed when mice were fully mature, resulted in exacerbated AD neuropathologies, such as APP deposition, tau aggregation, microglia activation and astrogliosis in brain tissues. The authors concluded that chronic inflammation induces AD-like pathologies in mice in an age-dependent manner. The streptozotocin-induced neuroinflammation model relies on peripheral injection of the glucosamine-nitrosourea compound streptozotocin (STZ). STZ induces oxidative stress and impairs brain glucose metabolism associated with insulin signal transduction failure. This drug produces pancreatic insulitis and causes diabetes mellitus in mice (131). In addition, the intracerebroventricular injection of STZ in rats caused chronic inflammation (astrocytosis and microgliosis) accompanied by neuronal loss and neurodegenerative lesions in the brain (132, 133). In this model, neuroinflammation was detected 1-week post-injection and tau hyperphosphorylation and amyloid deposition were detected as early as 3 and 12 weeks, respectively (134). Finally, p25 and 3xTg transgenic mouse models are two additional and relevant neuroinflammatory models. First, p25 transgenic mice overexpress human p25, an activator of the cyclin-dependent kinase 5 (cdk5) that regulates cell cycle and plays a key role in brain development (135). In this model, astrogliosis and the increased expression of proinflammatory cytokines and chemokines (IL-1, TNF-α, TGF-β, and MIP-1α) were detected at 1 week of induction of p25 expression (136). Microgliosis, tau hyperphosphorylation and amyloid pathology were detected only at 4 and 8 weeks, and cognitive deficits were observed within 6 weeks after p25 induction. The authors suggested that in p25 transgenic mice, neuroinflammation is an early event in the pathogenesis of AD and is independent of β-amyloid and tau phosphorylation. Second, the triple transgenic mouse of AD (3xTg) is the only transgenic model to express three major genes (PS1_M146V_, APP_Swe_, and tau_P301L_) associated with EOAD and behavioral and neurological changes that are observed in the human form (137, 138). This model exhibits both extracellular Aβ deposits and hyperphosphorylated tau tangles. In addition, it shows CNS inflammation such as astrogliosis, activated microglia and neurodegeneration including synapse loss, already at pre-symptomatic stage (5–20 post-natal weeks) (139). Systemic injection of polyI:C exacerbated neuroinflammation and AD pathology in 3Txg mice (130). Overall, these two neuroinflammatory mouse models provide similar chronological progression of AD pathologies as seen in humans. Therefore, they are considered suitable models to study the inflammatory hypothesis of AD and to discover effective therapeutic agents.

So far in mouse AD models, therapeutic approaches targeting different components of the neuroinflammatory response have shown promising results. As microglia activation is a key step in the neuroinflammatory pathogenesis of AD, depletion of these cells in adult mice has been shown to significantly reduce tau progression (140). Recently, Spangenberg et al. demonstrated the elimination of more than 95% of microglia in an AD mouse model using the drug PLX3397. This molecule inhibits colony-stimulating factor 1 receptor (CSF1R), which microglia need to survive. Feeding animals with this orally bioavailable and brain-penetrant inhibitor resulted in the reduced accumulation of Aβ inside neurons and prevented the formation of neuritic plaques in the brain (141). Moreover, cromolyn sodium, an FDA-approved drug for the treatment of asthma, has been shown to inhibit Aβ aggregation both *in vitro* and *in vivo* (142). This drug also has proved successful in shifting microglia from a proinflammatory/neurotoxic to a pro-phagocytic/neuroprotective activation state in transgenic mice, thus enhancing efficient uptake of Aβ (143). Finally, specific targeting of TLR2 has been proposed to be an important step for the attenuation of microglia activation. Indeed, neutralizing antibodies against TLR2 blocked Aβ-induced expression of proinflammatory cytokines in mouse primary microglia (144). TLR2 involvement in microglia activation was further confirmed using TLR2 deficient mouse microglia. In this study, the authors suggested that Aβ peptide binds to the TLR2 receptor expressed on microglia and primed these cells to a proinflammatory state via TLR2 signaling pathway activation. The α-melanocyte-stimulating hormone (MSH), a neuropeptide and member of the melanocortin family, has demonstrated anti-inflammatory properties in the treatment of CNS inflammatory disease such as experimental autoimmune encephalomyelitis (EAE) (145). Treatment of primary rat microglia cultures with an analog of MSH, the synthetic peptide NDP-MSH, resulted in the inhibition of TLR2- and TLR4-mediated TNF-α release (146). In addition, NSD-MSH treatment reduced the levels of Aβ and tau phosphorylation, inflammation, neuronal apoptosis, and improved cognitive functions in 3Txg transgenic mice compared to control groups (147). All together, these findings suggested that NDP-MSH promotes the development of microglia into an anti-inflammatory M2-like phenotype. Potentially, this molecule could be beneficial in preventing the neuroinflammation associated with AD. While anti-inflammatory therapy, such as NSAIDs, has been shown to reduce AD pathology in animal models, it has not been proven to be effective in human clinical trials (148–150). However, the inhibition of specific proinflammatory cytokines represents a more promising therapeutic strategy for AD. Blocking antibodies that bind to IL-1β have been shown to attenuate tau pathology and microglia activation in the brain of 3Txg AD mice (151). In addition, the administration of a monoclonal antibody against TNF-α (Infliximab) has been demonstrated to reduce amyloid plaques and tau phosphorylation in APP/PS1 transgenic mice (152).

### HSV1-Induced Neuroinflammation

So far, most studies to investigate neuroinflammation in AD pathogenesis have focused on understanding the mechanisms underlying glia cell activation and downstream inflammatory events in the late stages of AD (e.g., Aβ accumulation). Very little attention has been given to how glia cells initially become activated and whether in the early stages of AD, an infectious agent may trigger their activation.

The role of HSV1 infection in the activation of glia cells has been well-studied in the context of HSE. HSV1-infected primary microglia and astrocytes produce high levels of proinflammatory cytokines, including IL-6 (153, 154). Moreover, the anti-inflammatory cytokine IL-10 inhibits the production of HSV1-induced inflammation in human microglia (155). These results suggest that microglia are key mediators in the neuroinflammatory response to HSV1 infection. Several *in vivo* studies reported that HSV1-induced inflammation in mouse microglia and astrocytes is mediated by TLR2 signaling pathway (156–158). Moreover, Marques et al. showed that microglia remained activated in the brain of HSV1-infected mice at 30 dpi, a time when neither infectious virus nor viral replication could be detected (159). The authors suggested that persistent microglia activation may contribute to neuronal damage and long-term neurological sequelae observed in HSE patients. Recurrent HSV1 infections of mice have been proposed to be one of the main causes of prolonged glia cell activation and neuroinflammation in the CNS, leading to long-term CNS damage (83, 84). Multiple HSV1 reactivation events in the brain induced gliosis and increased brain levels of IL-1β and IL-6. These events were also accompanied by the increased expression of neurodegenerative markers (e.g., Aβ and tau phosphorylation levels) and cognitive deficits. It was recently proposed that CNS asymptomatic HSV1 reactivation events can occur in recurrent HSV1 infections and lead to a mild and chronic inflammation of the brain with a non-fatal outcome. While microglia and astrocytes not only have pro-inflammatory properties, they also orchestrate the antiviral response to HSV1, such as the production of type I IFN (160, 161). The production of type I IFN in microglia and astrocytes is mediated by TLR3-dependent mechanisms (162). Defects in the TLR3-type I IFN signaling pathway were correlated with severe HSE in some patients (163). While a large number of humans are infected with HSV1, only a small minority ever experience HSE. This fact suggests that while glia cells efficiently contribute to antiviral defense in HSV1 infection, they cause limited CNS symptoms (from asymptomatic reactivations to mild HSE). However, even though most people experience recurrent HSV1 infections with no to mild symptoms, recurrent infections may actually might predispose them more to develop persistent inflammation in the brain and subsequent neurodegenerative diseases.

### Herpesvirus-Induced Neuroinflammation in the PNS: Where It All Starts?

A second aspect of the infectious hypothesis for AD is that the immune response to the infection must be centered in the brain. One possible complication is the fact that herpesviruses, such as HSV1, are primary invaders of the PNS, not the CNS. However, because the PNS and CNS are intimately connected, it may be that PNS responses to infection will have direct and indirect effects in the CNS. This possibility should be further examined in regards to AD pathogenesis. Several animal models of peripheral HSV1 infection already exist such as the rabbit and mouse eye models and the guinea pig models (164, 165). These models have been widely used to study multiple aspects of HSV1 biology, including viral reactivation in the PNS (166–170). However, they have rarely been used to investigate the direct role of herpesvirus-induced neuroinflammatory responses in the PNS in initiating AD in the brain.

Recently, Laval et al. dissected the mechanisms of herpesvirus-induced neuroinflammatory responses in the PNS *in vivo* and its direct impact on the CNS (171, 172). This work was done using pseudorabies virus (PRV), an alpha herpesvirus distantly related to HSV1. PRV has been used extensively to study the mechanisms of neuronal spread from the site of primary infection to the PNS and CNS, neuro-circuitry, and immune responses to herpesvirus infections (173). PRV is a swine alpha herpesvirus, which can infect many other animal species, including mice and rats (174). PRV infection has much in common with HSV1 infection. Using a mouse footpad inoculation model, the authors demonstrated that a virulent strain of PRV induced a specific neuroinflammatory response (171). There was a strong correlation between the amount of infectious PRV detected in the PNS neurons and the production of two specific pro-inflammatory cytokines, IL-6, and granulocyte colony-stimulating factor (G-CSF) in a wide array of other tissues. Recently, Laval et al. further characterized the early events of the neuroinflammatory response to PRV infection in mice. They demonstrated that peripheral PRV infection primes PNS neurons to a state of inflammation very early after infection (within hours) (172). This priming resulted in an increase of proinflammatory cytokines (IL-6 and G-CSF) levels in the PNS and CNS simultaneously, and without active viral replication in the brain. They also showed that TLR2 and IFN type I play crucial roles in modulating this early neuroinflammatory response after peripheral infection. This long-distance immune signaling from PNS to CNS during herpesvirus infection was further supported by a study with vesicular stomatitis virus demonstrating that virus infection could activate long-distance interferon signaling in uninfected regions of the brain (175). The footpad inoculation model was useful for characterizing herpesvirus-induced neuroinflammatory responses *in vivo* because it was possible to separate local events at the site of infection and events in the PBS from those occurring in the CNS in both space and time. Infection in this model travels a greater distance from the periphery to the PNS and CNS neurons via the sciatic nerve and to the CNS via the spinal cord. As a result, it provides a clear assessment of viral kinetics and associated immunopathological processes initiated in the PNS and its potential role in long-distance immune-signaling to the CNS (176). These results provided a solid foundation for the role of peripheral herpesvirus infection in the global inflammatory priming of the brain. Future work should focus specifically on the role of HSV1-induced neuroinflammation in the PNS and its long-term neurological effects on the CNS.

## Conclusions and Future Perspectives

**Figure 1:**
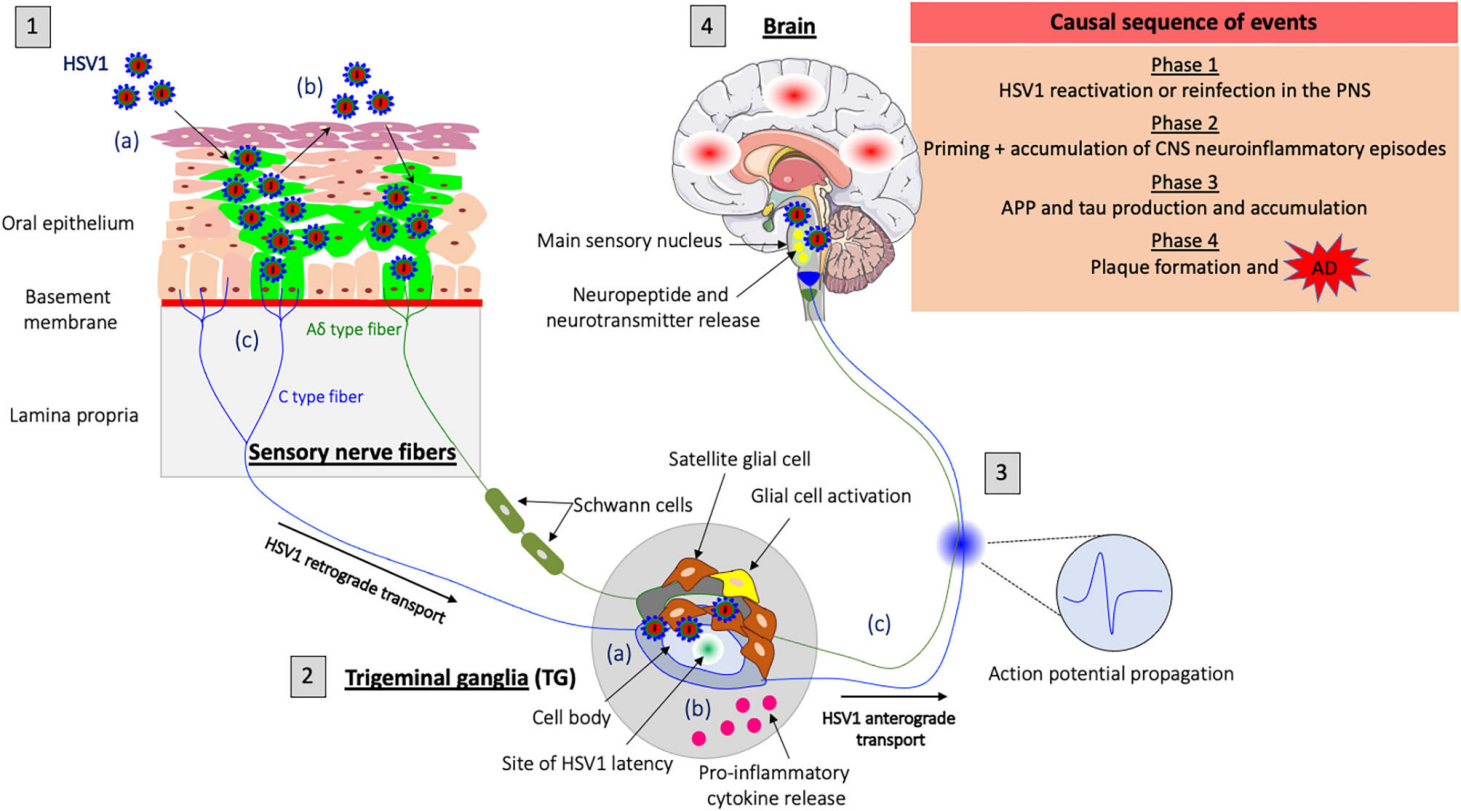
Model of HSV1-induced neuroinflammation in the PNS as a trigger for Alzheimer's disease (AD). **(1)** HSV1 first replicates in the oral mucosa epithelial cells; (a) HSV1 infection; (b) Viral spread within the mucosal epithelium and viral shedding; (c) HSV1 enters nerve endings of the PNS, including those coming from the trigeminal ganglia (TG) and spreads in the retrograde direction in axons to the ganglia. **(2)** HSV1 initiates a productive infection in TG neurons; (a) HSV1 replicates in cell bodies of TG neurons; (b) HSV1 induces a neuroinflammatory response (e.g., proinflammatory cytokine release and glial cell activation) in the PNS following primary infection or reactivation from latency; (c) New progeny virions can further spread in the anterograde direction and infect the CNS. **(3)** Synaptic transmission of the inflammatory response accompanied by the release of neuropeptides, neurotransmitters and the propagation of specific action potentials from the TG to the main sensory nucleus located in the brainstem. **(4)** Priming of the brain to a similar inflammatory state following transduction of inflammatory stimuli from the PNS. HSV1 reactivation or new primary infection of the PNS will thus enhance inflammatory responses in the brain. These events are likely to trigger the production of APP and tau proteins in the brain and repeated inflammation will lead to the accumulation of these proteins and the development of Aβ plaques that cause AD.

AD represents a major global health challenge with serious economical and personal impact on those affected and their families. Despite more than 3 decades of research on AD, there is no effective therapy to prevent or cure the disease. This fact makes it all the more necessary to better understand the pathogenesis of AD and design novel therapeutics. While the amyloid cascade hypothesis has been a central focus of attention, the idea that a common infectious agent could contribute to the development of AD is gaining support (the infectious hypothesis). There are two critical elements of the infectious hypothesis: (1) the infectious agent must be a common human infection and must persist in the host for years or must periodically infect the host and (2) the immune response to this agent involves the nervous system. HSV1 and its remarkable capacity to establish a reactivable infection in the nervous system is attracting more attention for a possible role in AD. It is noteworthy that treatment with specific anti HSV1 antiviral agents are reported to efficiently reduce AD pathology in both *in vitro* and *in vivo* systems. While HSV1 might a contributing factor in AD, additional factors (e.g., host and environmental factors) are likely to be involved in the sporadic manifestation of AD (177). Moreover, neuroinflammation has now emerged as an important component of AD pathogenesis. Several anti-inflammatory therapies have been tested and showed promising results in AD mouse models. Their efficacy in humans awaits clinical trials. The current infectious/inflammation hypothesis of AD suggests that recurrent HSV1 infections or periodic reinfections may cause persistent inflammation and long-term CNS damage leading to neurodegeneration. Reactivation of latent infections in the PNS may be sufficient to cause CNS inflammation without the presence of virus in the brain. Another idea is that latent infections can occur in the CNS and that reactivation of these latent infections may be important. While the neuroinflammatory response occurred before the appearance of Aβ plaques in the CNS, future work should also examine this response at earlier stages of the disease. Our lab demonstrated how herpesvirus infection of the PNS triggers specific inflammatory responses in both the PNS and CNS within hours post-infection. We therefore propose a model focused on HSV1 infection/reactivation in the PNS that, over time, provides the trigger for the initiation and development of AD (Figure 1). Targeting the PNS might represent a novel therapeutic approach to prevent AD. For instance, preventing HSV1 reactivation from PNS latency sites (e.g., TG) could efficiently stop the spread of new progeny virions from the TG to the brain. It might also reduce the priming of PNS neurons by infection and subsequent initiation of CNS neuroinflammation. Ultimately, this strategy might prevent the establishment of HSV1 latency and halt the development of AD pathology.

## Author Contributions

KL suggested the idea and wrote the review. LWE critically reviewed, corrected, and guided the completion of the review. All authors read and approved the final manuscript.

## Conflict of Interest

The authors declare that the research was conducted in the absence of any commercial or financial relationships that could be construed as a potential conflict of interest.
